# Coring Recanalization of a Chronically Occluded Left Subclavian Vein

**DOI:** 10.19102/icrm.2018.090605

**Published:** 2018-06-15

**Authors:** Sanoj Chacko, Sohaib Haseeb, Kevin A. Michael

**Affiliations:** ^1^Heart Rhythm Service, Kingston General Hospital, Queen’s University, Kingston, ON, Canada

**Keywords:** Coring technique, fluoroscopy, implantable cardioverter-defibrillator

## Abstract

A coring technique is an effective strategy to overcome short linear segmental chronic occlusions in subclavian veins caused by previously implanted leads. We present the case of an 82-year-old male who was transferred from a primary care center for consideration for implantable cardioverter-defibrillator placement following an episode of sustained monomorphic ventricular tachycardia and hemodynamic compromise that required direct-current cardioversion. A coring technique was successfully used in this patient.

## Introduction

**Figure 1:**
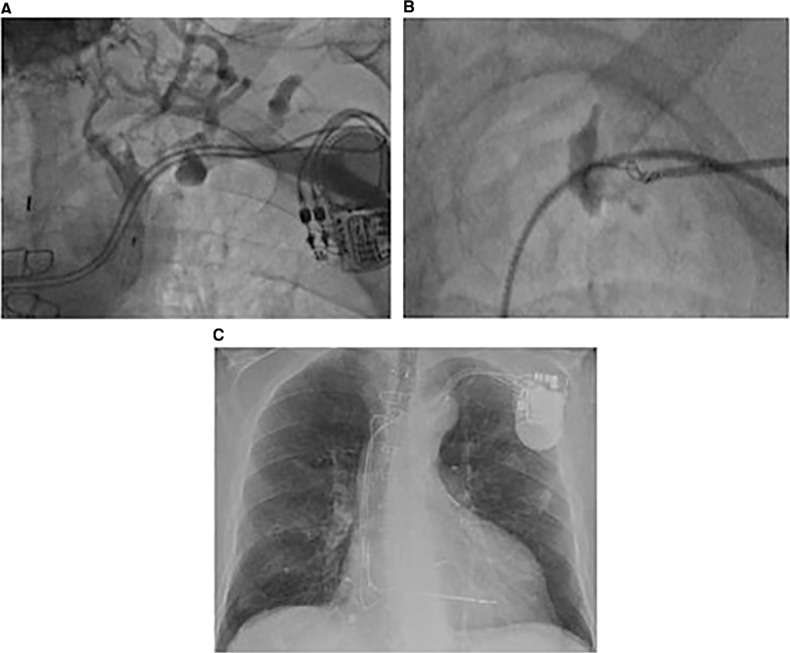
A composite of two cases showing the coring technique. **A:** A left upper extremity venogram showing an occluded subclavian vein with collaterals filling antegradely. **B:** A beveled edge needle in the subclavian vein distal to the stenosis with the Asahi Grand Slam angioplasty guidewire (Abbott Laboratories, Chicago, IL, USA) and contrast injection. **C:** Successful implantation of an ICD lead.

An 82-year-old male was transferred from a primary care center for consideration for implantable cardioverter-defibrillator (ICD) placement after experiencing an instance of sustained monomorphic ventricular tachycardia (MVT) and hemodynamic compromise that required direct-current cardioversion. He was known to have ischemic cardiomyopathy and triple vessel coronary artery disease, having undergone coronary bypass grafting in 1993. His electrocardiogram showed left bundle branch block morphology. The patient had undergone dual-chamber permanent pacemaker implantation 10 years prior for high-grade atrioventricular block and was pacing-dependent. His most recent echocardiogram had shown severe left ventricular systolic dysfunction with an ejection fraction of < 30%. He was on optimum heart failure therapy with New York Heart Association class I symptoms and was not clinically fluid-overloaded. Based on the current American College of Cardiology/American Heart Association (ACC/AHA) and Heart Rhythm Society (HRS) guidelines, the patient was deemed to meet the criteria to receive a cardiac resynchronization therapy defibrillator (CRT-D) upgrade. A preprocedure venogram of the left subclavian vein showed a totally occluded vessel with collaterals filling antegradely **(Figure 1A)**. The patient was noted to be right-handed and functionally active; he reported preferring to keep the device on his left side. Following discussion about the potential options, such as lead extraction and reimplantation and coring recanalization, the patient opted for the latter. Therefore, we proceeded with the device upgrade on the left side using a coring recanalization technique.

## Methods

The procedure was performed with a surgical backup while the surgeons were on-site. A beveled edge needle (7 cm/18-gauge; Cook Medical, Bloomington, IN, USA) was advanced under fluoroscopic guidance into the subclavian/axillary vein intersection over the first rib using the stored cine venogram as a scout image. A contrast mixture (50/50 saline contrast) was injected into the vessel through a side arm port to confirm a coaxial location with the lumen of the vein, and the tip of the puncture needle was then advanced into the occlusion. A high-support, nonhydrophilic (to avoid stripping of the coating) angioplasty guidewire (Asahi Grand Slam guidewire, 0.36 mm × 180 cm; Abbott Laboratories, Chicago, IL, USA) was used as a battering ram for advancement judiciously into the organized thrombus and was then pulled back into the needle. The needle was subsequently telescoped over the guidewire, following the course plotted by the guidewire, and was kept parallel to the existing leads **(Figure 1B)**. This maneuver was delicately repeated until the guidewire passed into the vessel lumen proximal to the stenosis. The Asahi Grand Slam guidewire (Abbott Laboratories) was confirmed on fluoroscopy as passing into the inferior vena cava and thus remaining intraluminal. The beveled needle was withdrawn and sequential short sheath dilators starting with the smallest caliber available [5-French (Fr)] were then used to dilate through the stenosis. Eventually, a 7-Fr splittable sheath (Peel-Away Introducer 7-Fr, 2.3 mm; Abbott Laboratories, Chicago, IL, USA) was admitted over the guidewire through the stenosis and a single-coil Durata™ lead (Abbott Laboratories, Chicago, IL, USA) was subsequently successfully implanted into the apical septum of the right ventricle **(Figure 1C)**. A further attempt to implant a left ventricular lead was not technically feasible; therefore, the CRT-D implantation was ultimately abandoned. The overall procedure time was two hours using 30 mL of contrast, with a fluoroscopy time of 21 minutes. The patient was discharged on optimum medical therapy and was stable at six months after the operation.

## Conclusion

A coring technique is an effective strategy to overcome short linear segmental chronic occlusions in subclavian veins caused by previously implanted leads. This method avoids accessing the contralateral side and removes the need for using subcutaneously implanted ICD systems when vascular access is difficult or unattainable. Balloon angioplasty of these chronic occlusions may be suboptimal as the site of occlusion is prone to recoil. By “coring” through the organized thrombus, a microchannel remains present to allow for the admission of an extra support wire.

